# Resizing the largest known extinct rodents (Caviomorpha: Dinomyidae, Neoepiblemidae) using occipital condyle width

**DOI:** 10.1098/rsos.220370

**Published:** 2022-06-15

**Authors:** Russell K. Engelman

**Affiliations:** Department of Biology, Case Western Reserve University, Euclid Avenue, 10900 Cleveland, OH, USA

**Keywords:** Rodentia, South America, body size, mass estimation, palaeontology, nonlinear allometry

## Abstract

Several extinct chinchilloid rodents in the clades Dinomyidae and Neoepiblemidae grew to sizes much larger than any living rodent species. However, the exact size of these rodents is a matter of controversy, with authors disagreeing due to issues over extrapolation and model selection. Prior estimates for the two largest extinct rodents, *Phoberomys pattersoni* and *Josephoartigasia monesi*, range from 230 to 700 kg for *P. pattersoni* and 350 to 2600 kg for *J. monesi*. Here, I estimate body mass in large, extinct rodents using occipital condyle width (OCW), a strong predictor of body size in mammals, using a dataset that circumvents many of the issues faced by previous studies of species. Body masses under shape-corrected OCW are much lower than previous studies: 108–200 kg for *P. pattersoni* and 480 kg for *J. monesi*. Mass estimates for other rodent taxa (*Neoepiblema*, *Telicomys*, *Dinomys*) agree with previous studies. Estimates using skull length, corrected condyle width and head-body length are similar, suggesting estimates of 150 kg for *Phoberomys* and 480 kg for *Josephoartigasia*, and that larger estimates of 700 and 1200 kg are unlikely. High estimates in previous studies appear to be due to the unrecognized, nonlinear relationship between certain skeletal measurements (skull size) and body mass.

## 1. Introduction

The fossil record is rife with examples of extinct species that are much larger than any of their modern relatives [1–4]. Exactly how large these animals were is necessary for reconstructions of palaeobiology and evolutionary history, as virtually every aspect of an organism's biology is influenced by body size [5–7]. However, estimating body size can be difficult in large, extinct taxa such as these, as it often requires extrapolating data beyond the range of body sizes spanned by extant taxa, which increases prediction errors [7,8]. Additionally, extinct members of clades may differ morphologically from their closest extant representatives in ways that cannot merely be attributed to size, thus making estimates from simple linear scaling relationships even more unreliable.

**Figure 1.**
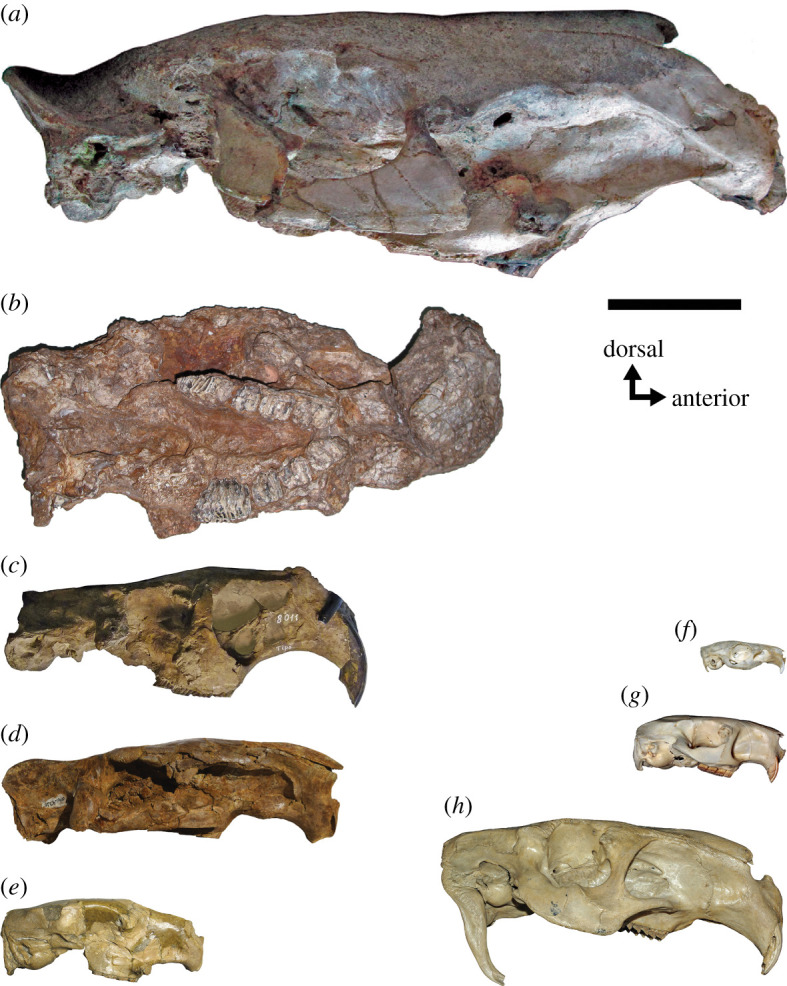
Skulls of giant extinct caviomorph rodents (*a*–*e*) in right lateral view (except *b*) compared with living caviomorphs (*f*–*h*). (*a*) *Josephoartigasia monesi* (MNHN 921, Dinomyidae, courtesy of A. Rinderknecht); (*b*) *Phoberomys pattersoni* (AMU-CURS 255, Neoepiblemidae, displayed in ventral view due to crushing, courtesy of J. Carrillo); (*c*) *Telicomys giganteus* (MACN 8011, Dinomyidae, modified from [22]) (*d*) *Neoepiblema acreensis* (UFAC 4515, Neoepiblemidae, modified from [24]); (*e*) *Tetrastylus intermedius* (MACN-Pv 8323, left lateral reversed, Dinomyidae, courtesy of M. Ercoli); (*f*) *Cavia porcellus* (D. Croft pers. coll.); (*g*) *Dinomys branickii* (FMNH 66891); and (*h*) *Hydrochoerus hydrochaeris* (CMNH 19121). Scale = 10 cm.

Consider the controversy over the size of the largest extinct rodents. The largest living rodent, the capybara (*Hydrochoerus hydrochaeris*, Caviidae)*,* can weigh up to 91 kg, with more typical weights ranging from 35 to 65.5 kg [9]. However, even larger rodent species are known to exist in the fossil record, most notably *Josephoartigasia monesi* (Dinomyidae) and *Phoberomys pattersoni* (Neoepiblemidae) (figure 1). Both *Josephoartigasia* and *Phoberomys* are represented by well-preserved cranial remains [10,11], and well-preserved postcranial remains are also known for *P. pattersoni* [12]. *Josephoartigasia* and *Phoberomys* pertain to separate but closely related clades among Chinchilloidea that appear to have evolved large body sizes independently [13,14] and are more broadly related to chinchillids and the modern pacarana (*Dinomys branickii*, Dinomyidae) among living rodents.

*Josephoartigasia* and *Phoberomys* were much larger than the extant *Hydrochoerus*, but exactly how large is a matter of controversy. Initial studies proposed body masses of approximately 700 kg (range of 436–741 kg) for *Phoberomys* [12] based on measurements of the humerus and femur and 1211 kg (range of 468–2586 kg) for *Josephoartigasia* [11] based on measurements of the skull, with these animals regarded as comparable to large bovids in size [15,16]. However, later studies have suggested smaller sizes of 234–250 kg [17,18], 420–580 kg [19] or 444–655 kg [13] for *P. pattersoni* (some of which are based on estimates from craniodental variables rather than postcranial ones) and sizes of 622–917 kg [13] or potentially as low as approximately 350 kg for *J. monesi* [8], respectively. Estimating body size in these taxa is made challenging by the fact that morphological features of these extinct rodents do not show consistent patterns of allometric scaling with living species. For example, compared with living rodents *Josephoartigasia* appears to have an elongated skull (based on the length of the molar row and other craniodental metrics relative to overall skull size; [8,20]) and the femur of *Phoberomys* is disproportionately robust relative to its overall size [18]. This makes it difficult to find which best variable or combination of variables most accurately estimate body size.

The challenges in estimating the body mass of extinct giant caviomorphs like *Phoberomys* and *Josephoartigasia* primarily stem from three issues. First, these animals are so much larger than any of their close living relatives (i.e. extant rodents) that estimates using models derived only from rodents are potentially prone to significant extrapolation error [7,8]. Second, some of the skeletal measurements previously used to estimate body mass in these taxa may capture unique patterns of anatomical variation not seen in their living relatives, and thus mass estimates using these methods may be unreliable. Third, the largest extinct caviomorphs are often only known from incomplete remains, preventing the use of volumetric methods [21,22]. Thus, what is needed is a method of estimating body mass that can be applied to extinct caviomorphs, is as independent of species-specific patterns of anatomy as possible and can include data from a wide variety of mammals that can be better used to constrain body size estimates in giant extinct caviomorphs. Millien [8] suggested that this would be the only way to resolve the controversy surrounding the size of taxa like *Josephoartigasia* and *Phoberomys*.

One potential metric that might fit these criteria is occipital condyle width (OCW). Engelman [23] recently found that OCW closely correlated with body mass across a broad sample of therian mammals. Notably, this study also found that although there is a very strong correlation between OCW and body mass, this correlation is not strictly linear even after log transformation; instead showing a log-allometric relationship. The study also found a similar relationship between skull length and body mass in mammals [23]. More specifically, there are several aspects of OCW which make it a strong candidate for estimating body mass in extinct caviomorphs. First, due to its position at the joint between head and body, OCW appears to scale with the size of the postcranium, despite being a cranial measurement [23]. This circumvents the issue of estimating body mass on cranio-dental features if the animal has a large head relative to its postcranium [23], as is potentially the case with large extinct rodents [8,24–26], but is still a measurable feature in species like *Josephoartigasia* where the postcranium is unknown. Secondly, because OCW accurately predicts body mass across nearly all therian mammals, it is possible to use this measurement to more accurately estimate body mass in giant extinct caviomorphs like *Phoberomys* and *Josephoartigasia*, because it is possible to include data from other large mammals in the same approximate size range as these extinct caviomorphs (i.e. ungulates, carnivorans) and thus the model can make predictions of body mass without the need for extrapolation. Here, I use the OCW dataset of Engelman [23] to estimate the body mass of *Phoberomys*, *Josephoartigasia,* and several other closely related extinct giant rodents and discuss these estimates within the context of the debate over these animals' body size.

## 2. Material and methods

Body masses in the extinct giant caviomorph rodents *Phoberomys pattersoni* (Neoepiblemidae) and *Josephoartigasia monesi* (Dinomyidae), several closely related taxa in Neoepiblemidae (*Neoepiblema acreensis*) and Dinomyidae (*Arazamys castiglionii*, *Telicomys giganteus* and *Tetrastylus intermedius*), and two specimens of the sole extant dinomyid *Dinomys branickii* were estimated using the regression equation between the natural logarithms of body mass and OCW generated using a sample of species-average weights for 404 taxa derived from 2127 individual specimens [23]. One of these specimens of *D. branickii* (CMNH 22121) had an associated body mass of 9.525 kg, helping to constrain mass estimates. The only change to the dataset of Engelman [23] was the values for *D. branickii* were updated based on remeasurement of CMNH 22121. In Engelman [23], 96 taxa are rodents, a much larger sample than previous studies that attempted to calculate body mass for *Phoberomys* and *Josephoartigasia* [8,11,17,18]. All analyses were performed using R 4.1.1 [27], see electronic supplementary material, Information for more details on statistical analyses. Body masses were estimated using the all-mammal regression equations of Engelman [23], rodent-specific regression equations from that study were considered less optimal here (but the results of which are reported in the electronic supplementary material, Information) to avoid the same problem of extrapolation as in previous studies [8] and because the residuals of the rodent-specific regressions in Engelman [23] were non-normally distributed.

Extinct caviomorphs were either measured from photographs or taken from the previously published literature (see tables 1 and 2). Body mass was estimated under several different models, including ones without making assumptions of morphology as well as ones using a correction factor assuming the extinct rodent taxa deviate from the regression line to a similar degree as the extant *Hydrochoerus* and *Dinomys* (calculated by multiplying the log-transformed estimated body mass for the extinct rodents by 1 plus the mean residual value for these two extant taxa before exponentiating). Given that *Phoberomys* and *Neoepiblema* have strongly ‘rabbit-like’ condyles (*sensu* [23]), body mass in these taxa was also estimated using the multivariate equation of Engelman [23] treating occipital condyle shape as an additional categorical variable.

**Table 1.** Body mass estimates and 95% prediction intervals for *Josephoartigasia monesi*, *Phoberomys pattersoni* and other large chinchilloid caviomorphs using occipital condyle width (OCW). OCW, occipital condyle width; PAR, width of OCW plus paracondyles (in Dinomyidae); BM_OCW_, body mass estimated using OCW with no correction for morphology; BM_COR_, body mass estimated using OCW assuming a similar deviation from the regression line as *Dinomys* and *Hydrochoerus*; BM_PAR_, body mass estimated using OCW including paracondylar width assuming a similar degree of overestimation as in *Dinomys*; BM_LAG_, body mass controlling for shape assuming large caviomorphs have condyles similar to Lagomorpha (see [23]). All linear measurements in mm and all body masses in kg. * = known body mass of 9.5 kg. For additional estimates see electronic supplementary material, Information.

taxon	specimen	Ref.	OCW	PAR	BM_OCW_	BM_COR_	BM_PAR_	BM_LAG_
*Josephoartigasia monesi*	MNHN 921	[11]	67.0	113.7	145.8 (67.7–313.9)	254.0 (118–546.7)	482.7 (223.8–1041.4)	296 (147.4–596.0)
*Phoberomys pattersoni*	UNEFM-VF-020	[12]	51.6	—	63.0 (29.3–135.6)	109.8 (51.0–236.1)	—	127.0 (63.3–255.0)
*Phoberomys pattersoni*	CIAAP 1438	[28]	51.4	—	62.3 (28.9–133.9)	108.4 (50.4–233.2)	—	126.0 (62.5–252.0)
*Phoberomys pattersoni*	AMU-CURS 161	[10]	approximately 54	—	73.0 (34.0–157.1)	127.2 (59.1–273.6)	—	148 (73.4–296)
*Arazamys castiglionii*	MNHN 2521	[29]	approximately 55	107.9	77.5 (36.0–166.7)	135.0 (62.8–290.4)	411.5 (190.8–887.6)	157 (78.0–315)
*Dinomys branickii*	CMNH 22121*	[23]	26.23	33.8	6.5 (3.0–14.1)	11.4 (5.3–24.5)	10.0 (4.6–21.5)	13 (6.5–26)
*Dinomys branickii*	FMNH 152061	[24]	26.68	34.2	6.9 (3.2–14.9)	12.1 (5.6–26.0)	10.3 (4.8–22.2)	14 (6.9–28)
*Neoepiblema acreensis*	UFAC 4515	[30]	39.78	—	26.8 (12.5–57.7)	46.7 (21.7–100.5)	—	54 (26.8–108)
*Telicomys giganteus*	MACN 8011	[31]	46.62	—	45.3 (21.1–97.4)	78.9 (36.7–169.6)	—	91 (45.4–183)
*Tetrastylus intermedius*	MACN-Pv 8323	[24]	27.33	42.2	7.5 (3.5–16.2)	13.1 (6.1–28.2)	20.8 (9.7–44.8)	15 (7.5–30)

**Table 2.** Body mass estimates and 95% prediction intervals for *Josephoartigasia monesi*, *Phoberomys pattersoni* and other large chinchilloid caviomorphs using skull length (= condylobasal length, CBL), and estimated head-body length (HBL) assuming similar head-body proportions to *Dinomys* and *Hydrochoerus*. Abbreviations: BM_CBL_, body mass estimated using condylobasal length; BM_HBL_, body mass estimated using head-body length; BM_THR_, body mass estimated using multivariate regression of all three equations. All linear measurements in mm and all body masses in kg. * = known body mass of 9.5 kg. For additional estimates see electronic supplementary material, Information.

taxon	specimen	Ref.	CBL	HBL	BM_CBL_	BM_HBL_	BM_THR_
*Josephoartigasia monesi*	MNHN 921	[11]	498	2628	530.4 (208.6–1348.8)	576.0 (248.6–1334.6)	299.8 (165.1–544.6)
*Phoberomys pattersoni*	AMU-CURS 255	[10]	356	1879	200.9 (79.1–510.1)	212.3 (91.8–491.3)	—
*Phoberomys pattersoni*	AMU-CURS 161	[10]	336	1773	169.5 (66.8–430.2)	178.6 (77.2–413.2)	117.1 (64.7–211.9)
*Dinomys branickii*	CMNH 22121*	[23]	141	744	11.8 (4.6–29.8)	13.5 (5.8–31.1)	9.3 (5.1–16.7)
*Dinomys branickii*	FMNH 152061	[24]	142	751	12.1 (4.8–30.7)	13.9 (6.0–32.0)	9.7 (5.4–17.5)
*Neoepiblema acreensis*	UFAC 4515	[30]	264	1393	82.6 (32.6–209.5)	87.2 (37.7–201.5)	49.2 (27.2–89.2)
*Telicomys giganteus*	MACN 8011	[31]	271	1430	89.3 (35.2–226.6)	94.2 (40.8–217.8)	66.5 (36.8–120.2)
*Tetrastylus intermedius*	MACN-Pv 8323	[24]	164	866	19.1 (7.5–48.3)	21.2 (9.2–48.9)	12.6 (7.0–22.8)

**Figure 2.**
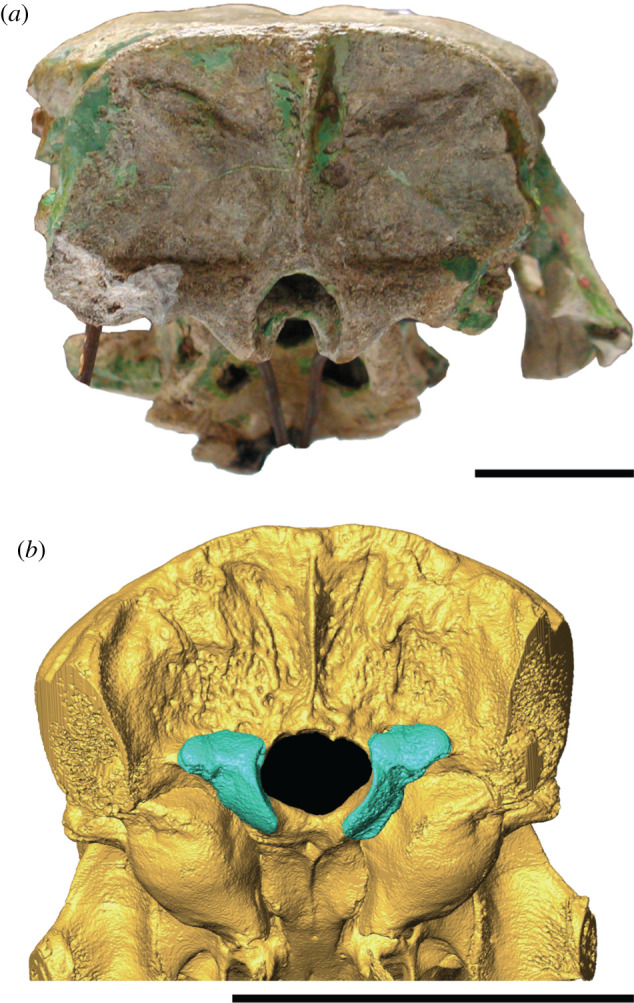
Skulls of *Josephoartigasia monesi* (MNHN 921, *a*) and *Dinomys branickii* (CMNH 22121, *b*) in posterior view, showing the morphology of the paracondyles in these species. (*b*) is a reconstructed CT image of this specimen with the occipital condyles and paracondyles highlighted in teal. Scale = 5 cm.

One concern about using OCW to estimate body mass in dinomyids is that dinomyids (but not neoepiblemids like *Phoberomys*) have large structures called paracondyles (figure 2) that project laterally from the occipital condyles onto the surface of the occiput, a dinomyid synapomorphy [24,29]. Some dinomyids, like *Josephoartigasia*, have extremely wide paracondyles (figure 2*a*). However, paracondyle presence and size does not appear to correlate with head or body size within dinomyids, and if paracondyles are included as part of OCW the occipital condyles of dinomyids are recovered as disproportionately wider than expected relative to the animal's size. Therefore, body mass estimates for dinomyids were examined both including and excluding paracondyles as part of OCW.

Additionally, body mass was estimated in fossil rodent taxa with known complete skulls using condylobasal length (CBL, often measured as condyloincisive length in rodents, measured from the anterior border of the upper incisors or their alveoli to the posterior margins of the occipital condyles), estimated head-body length (HBL, assuming a similar head-body ratio as in *Dinomys* and *Hydrochoerus*), and a multivariate equation treating all three variables together, using equations calculated from the broader all-taxon taxonomic dataset of Engelman [23] as an independent test of body size for these rodents.

The equations for OCW and skull length used in this study are not log-linear, but instead log-allometric. This is because the relationship between OCW and body mass or skull length and body mass does not form a straight line even after log-transformation, but instead shows nonlinear allometry [23]. Specifically, larger mammals have longer skulls and wider OCW relative to their body mass at larger body sizes, and plotting the log-transformed variables against one another shows a slightly curvilinear distribution. This interpretation is robust to testing: log-quadratic equations for these relationships produces significantly better model support statistics than log-linear ones (e.g. AIC, significance of a quadratic term), residuals versus fits plots are nonlinear, and the slopes of the relationship between the log-transformed independent variable and body mass becomes increasingly shallower at larger body sizes (see [23]).

Institutional Abbreviations. AMU-CURS, Alcaldía del Municipio de Urumaco, Falcón, Venezuela; CIAAP, Centro de Investigaciones Antropológicas Arqueológicas y Paleontológicas, Universidad Nacional Experimental Francisco de Miranda, Coro, Venezuela; CMNH, Cleveland Museum of Natural History, Cleveland, USA.; FMNH, Field Museum of Natural History, Chicago, USA.; MACN**,** Museo Argentino de Ciencias Naturales, Buenos Aires, Argentina; MNHNM, National Museum of Natural History, Montevideo, Uruguay; UFAC, Universidade Federal do Acre (Campus Rio Branco), Rio Branco, Brazil; UNEFM-VF, Universidad Nacional Experimental Francisco de Miranda, Vertebrate Paleontology Collection, Coro, Venezuela.

## 3. Results

Uncorrected OCW produces body mass estimates of 63–73 kg for *Phoberomys* (95% prediction interval (P.I.) = 28.9–157.1 kg, depending on the specimen) and 146.0 kg (95% P.I. = 67.7–319.9 kg) for *Josephoartigasia* (table 1), much lower than even the lowest estimates in previous studies. However, *Phoberomys* and *Josephoartigasia* exhibit a condition similar to *Dinomys* and *Hydrochoerus* in which the condyles are tall but narrow (similar to rabbits and dipodomyines, though more robust) [23]. Taxa with this kind of condylar morphology form a line parallel to the least fit line for the entire sample, suggesting the error in the body mass estimate is due to differences in condylar shape, rather than differences in allometry, and therefore OCW can still be used to estimate body mass in these taxa if differences in condylar shape are corrected for [23]. Correcting for differences in shape using a correction factor derived from *Dinomys* and *Hydrochoerus* produces body mass estimates of 108–127 kg (95% P.I. = 50.4–273.6 kg) for *Phoberomys* and 254 kg (95% P.I. = 118.0–546.7 kg) for *Josephoartigasia* (table 1), still low but closer to previous estimates.

**Figure 3.**
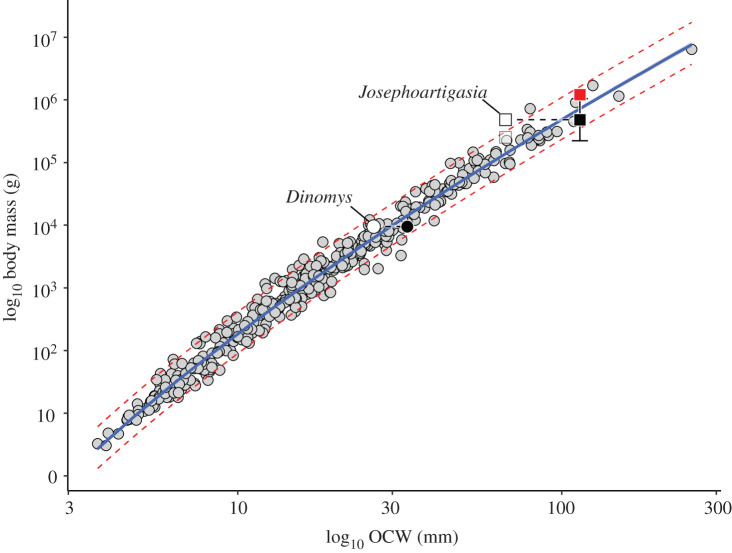
Plot of log_10_ OCW versus log_10_ body mass, showing the effect of including paracondyles on mass estimation in Dinomyidae. Circles represent specimen of *Dinomys branickii* with known weight (CMNH 22121), whereas squares represent *Josephoartigasia monesi*. White symbols represent position of taxon excluding paracondyles, whereas black symbols represent position when condyles are includes as part of OCW. Red square represents mean body mass estimate of *J. monesi* (1211 kg) from [11]. Translucent square represents estimated body mass of *J. monesi* using OCW excluding paracondyles but including correction factor.

Even if paracondyles are included as part of OCW, *Josephoartigasia* is estimated as having a lower mass than in previous studies (757 kg, 95% CI = 351–1633 kg). However, estimating body mass using paracondylar width in the specimen of *Dinomys branickii* with known mass results in OCW overestimating the weight of this individual by over 50% (figure 3). *Josephoartigasia* has proportionally wider and more robust paracondyles than *Dinomys* (figure 2), as is typical of eumegamyine dinomyids [25,29]. Therefore, including paracondyles in calculations of OCW probably overestimates body mass, agreeing with the observations of Álvarez and Ercoli [24]. Assuming paracondyles inflate body mass estimates to a similar degree as in *D. branickii*, this results in an estimated mass of 483 kg (95% P.I. = 224–1041 kg) for *J. monesi* (table 1).

Neoepiblemids lack paracondyles, but show an occiput morphology very similar to extant *Hydrochoerus* (at least, more so than dinomyids). This suggests that it might be more appropriate to use the regression model treating neoepiblemids as having ‘rabbit-like’ condyles (as in *Hydrochoerus*), rather than relying on comparisons with only *Hydrochoerus* and *Dinomys* where the apomorphic occiput of *Dinomys* may distort estimates. Doing so produces estimates of 125–150 kg (95% P.I. ranges from 62.5 to 296 kg depending on specimen) for *Phoberomys pattersoni*, and 54.0 kg (95% P.I. = 26.8–108 kg) for *Neoepiblema acreensis*, slightly higher than using the correction factor calculated based on *Dinomys* and *Hydrochoerus* (table 1, see also electronic supplementary material, Information).

The results using CBL and HBL agree with those of shape-corrected OCW. Skull length predicts a body mass estimate of 170–200 kg (66.8–510.1 kg) for *Phoberomys* and 530 kg (208.6–1348.8 kg) for *J. monesi* (table 2). The extreme upper end of the 95% prediction interval overlaps with the 1000+ kg estimate for *J. monesi* in previous studies [11], but prediction intervals in most regressions of body mass in mammals tend to be relatively large due to issues with log-transformation [32,33], and prediction intervals for CBL are greater than for OCW. It is also important to note the anatomical assumptions that the larger body masses (800+ kg) within the 95% prediction interval entail. Specifically, taxa in the upper tails of the 95% prediction interval for the CBL equation are all species with very small heads relative to body size such as felids and macropodids. Thus, large body mass estimates would only be possible if *Josephoartigasia* and *Phoberomys* had very small skulls relative to body size, which is unlikely given most large hystricomorphs such as *Dinomys* and *Hydrochoerus* have large heads [24,25], and extinct giant caviomorphs generally show small occiputs relative to skull size. Therefore, it is unlikely that the body masses at the extreme upper ends of the 95% prediction interval are plausible for *Josephoartigasia*.

For HBL, assuming the skull made up a similar percentage of head-body length as *Hydrochoerus* or *Dinomys* (in which the skull is 18–20% total HBL), then *Phoberomys* is predicted to have weighed 179–212 kg (95% P.I. = 77.2–491.3 kg, depending on the specimen) and *Josephoartigasia* is predicted to have weighed 576 kg (248.6–1334.6 kg), respectively (table 2). However, the agreement between CBL and HBL should not be taken to indicate that these metrics are more accurate than OCW, given that HBL in this study is calculated based off CBL, and dinomyids (and possibly neoepiblemids) appear to have relatively larger heads than expected for their size [13]. Using all three variables together produces values of 117.1 kg (64.7–211.9 kg) for *Phoberomys* and 299.8 kg (95% P.I. = 165.1–544.6 kg) for *Josephoartigasia*. Including paracondyles as part of OCW in the multivariate equation but assuming paracondyles influence OCW to a similar degree as *Dinomys* results in an estimated mass of 486.5 kg (95% P.I. = 211–1121 kg) for *Josephoartigasia*.

The two specimens of *Dinomys branickii* examined produced body mass estimates very close to reported values for this species [34,35]. In the specimen with recorded weight (CMNH 22121), corrected OCW produced an estimate very close to the actual mass, but given that this specimen was one of those used to calculate the correction factor this is to be expected. Skull length using a log-allometric model overestimated body mass in CMNH 22121, but this is to be expected as *Dinomys* has been considered by previous authors to have a relatively large head compared with its body size [24,34]. However, it does suggest that skull length might be expected to overestimate body mass in extinct dinomyids and neoepiblemids.

The body mass estimates for the other giant chinchilloids estimated here roughly agree with those in the previously published literature. The estimate of 54 kg treating OCW as ‘rabbit-like’, and 82.6 kg using skull length for *Neoepiblema acreensis* are close to the estimate of 79.95 kg in Ferreira *et al.* [36] and the volumetric estimates of 65–77 kg in Kerber *et al.* [21], though the estimate of 46.7 kg using corrected OCW is slightly low. However, it is much lower than the average mass of 159 kg estimated using the diameter of the humerus or femur in Kerber *et al.* [21]. This is very similar to the pattern seen in *Phoberomys*: previous studies estimating body mass using limb bone diaphyseal dimensions [12,18,19] produce much higher estimates than other skeletal proxies such as tooth row length (e.g. [17,18], OCW and CBL in this study), often producing estimates that are more than twice the size of estimates based on other skeletal variables. For *Telicomys giganteus*, the estimate of 78.9 kg using corrected OCW and 89.3 kg using skull length agree with previous estimates [13,31], which span a very wide range but cluster around 90–100 kg. For *Arazamys castiglionii*, the present body mass estimate of 411.5 kg is very similar to the estimates that Vucetich *et al.* [13] produced using the length of the upper dental series (P4–M3) assuming geometric similarity with other caviomorph taxa. Vucetich *et al.* [13] produced an estimated body mass of 309.4 kg when using the proportions of *Lagostomus maximus*, and 455.8 kg when estimating body mass assuming similar proportions to *D. branickii*. No prior body mass estimates are available for *Tetrastylus intermedius*. The fact that the methods used here produce values in *N. acreensis* and *T. giganteus* similar to those in previous studies and values in *D. branickii* similar to actual weights for the species suggest that the formulae for corrected OCW, skull length, etc. used here should produce relatively accurate estimates of body mass in *Phoberomys* and *Josephoartigasia*.

## 4. Discussion

**Figure 4.**
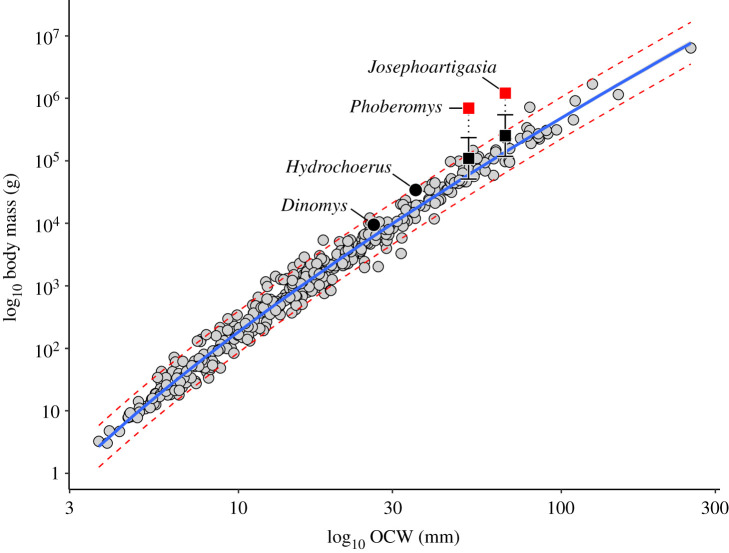
Scatterplot of log_10_ OCW versus log_10_ body mass, showing the positions of *Josephoartigasia* and *Phoberomys* assuming previous mass estimates of 700 and 1211 kg, respectively, are correct (represented by red squares), as well as the body mass estimated using OCW and the correction factor calculated from *Dinomys* and *Hydrochoerus* (represented by black squares). Black circles represent the position of *Dinomys* and *Hydrochoerus* based on extant individuals with known body mass.

Body mass estimates for *Josephoartigasia monesi* and *Phoberomys pattersoni* here are much lower than in previous studies, only 254–576 kg for *J. monesi* and 108–200 kg for *P. pattersoni*. By contrast, point estimates for other dinomyids and neoepiblemids using OCW (as well as HBL and log-allometric CBL) agree with previous studies, suggesting these methods should produce accurate results in larger taxa. Even after correcting for differences in occiput shape, and thus biasing estimates in favour of heavier body masses as much as could be mathematically justified, mass estimates for *Josephoartigasia* and *Phoberomys* using OCW (including their prediction intervals) only approached the lower end of mass estimates of later studies, suggesting that these taxa are much smaller than previously proposed. Similarly, even when including paracondyles in OCW, which in theory should produce overestimates of body mass based on comparisons with *Dinomys*, estimated body masses for *Josephoartigasia* are on the low end of previous studies. In order for previous body mass estimates of 700 and 1211 kg to be accurate, *Phoberomys* and *Josephoartigasia* would have to exhibit an OCW prediction error (PE) at least 378% (in *Josephoartigasia*) or 540% (in *Phoberomys*), much higher than that of any mammal in the extant dataset, including those with unusual occiput morphologies like *Dinomys* or *Hydrochoerus* (figure 4). The most extreme %PE in the extant sample of Engelman [23] is only 190% of the actual value, and these are from species represented by single individuals that due to context (being a captive *Camelus bactrianus* and an Alaskan *Lepus othus* collected in the middle of winter) might be expected to be overweight relative to their OCW-predicted body mass.

Notably, in the context of the present study, it is highly unlikely that *Josephoartigasia*, *Phoberomys* and other large caviomorphs had a very large body, a large skull relative to its body (as in *Dinomys*), and disproportionately small condyles. This is because the stability of the occipito-atlantal joint is critical for survival, as dislocation of the joint often leads to severing of the spinal cord and instant death [37]. A smaller occiput would result in a less stable joint because of the smaller articular surface area and increased torque from more closely positioned occipital condyles. This can be seen to some degree in taxa like *Hydrochoerus*, *Phoberomys* and lagomorphs: although these taxa have mediolaterally narrow occiputs, the occipital condyles are also opisthobasally long, which would be expected to keep articular surface area constant despite changes in shape.

The 95% prediction intervals of these estimates are very large, but this is a consequence of estimating body masses on a unitless, log-transformed scale and then converting the estimated values back to the arithmetic scale of interest (i.e. g or kg) by taking the antilog. This tends to produce extremely large and often mathematically impractical prediction intervals for biological data (e.g. [23,32,33,38]) due to detransforming long tails of distributions, even when the variable of interest strongly correlates with body mass [32]. Identifying a potential mathematical alternative to the extremely large prediction intervals seen in (e.g. using %PE or %SEE to calculate prediction intervals, which measure how far the average predicted, detransformed body mass differs from the actual value [38] and has been used to calculate prediction intervals in previous studies [18,39]) is beyond the scope of the study.

**Figure 5.**
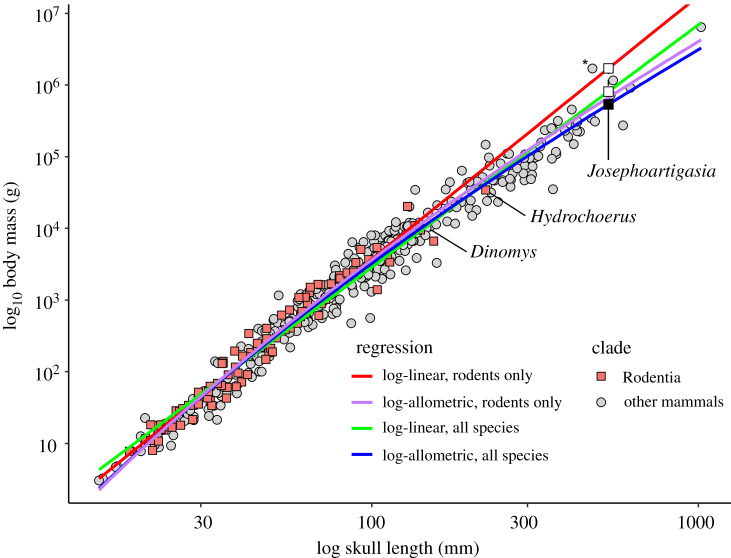
Plot of log_10_ skull length versus log_10_ body mass in mammals, showing how the log-linear rodent-only equation results in a dramatic overestimate of body mass in *Josephoartigasia* compared with all other models (see black and white squares). Data point with asterisk is specimen of *Bison bonasus* whose reported weight is a likely lapsus based on comparison with literature values [40].

Prior controversies surrounding very high mass estimates of *Josephoartigasia* [8,11] may be the result of failing to account for nonlinear allometry when estimating body mass. As mentioned in Methods, the relationship between skull length and body mass does not appear to be strictly log-linear in mammals, but log-allometric. Over relatively short spans of body size, deviations from normality due to this nonlinear allometry are negligible, but when dealing with clades that span very large ranges of body sizes, like rodents [12], these issues become increasingly pronounced. With the present data, if the body mass of *J. monesi* is estimated using a log-linear equation containing only rodents, it produces an estimated mass of 1330 kg (95% CI = 575–3072 kg, see electronic supplementary material, Information) (figure 5), relatively similar to the estimates Rinderknecht and Blanco [11] and Millien [8] produced using skull length. This is because most rodents are relatively small, resulting in a steeper slope relative to the curvilinear pattern of the data, but it greatly overestimates body mass in *Hydrochoerus* and *Dinomys*. Using a log-linear regression model with all mammals produces a body mass estimate of 686 kg (95% CI = 253–1860 kg) for *J. monesi*, much lower than Rinderknecht and Blanco's [11] estimate using skull length though higher than most of the estimates in the present study. Accounting for the curvilinear scaling pattern of the data using a log-allometric model, the resulting body mass is only 530 kg (95% CI = 209–1349 kg). A log-quadratic model produces relatively similar results to the log-allometric model used here (502.6 kg, 95% CI = 195.9–1289 kg). It is likely that this nonlinear relationship was not recognized by previous studies because the extant datasets used to estimate the body mass of *Josephoartigasia* and *Phoberomys* were composed solely of rodents: very few rodents are greater than 1 kg in weight and this obscures the slight curvature of the data that can be seen when comparing skull length versus body mass in all mammals (figure 5).

It is not clear what produces the log-allometric relationship between skull length and body mass, but one possibility is craniofacial evolutionary allometry [41], which causes larger mammals to have disproportionately longer rostra. This is somewhat supported by the results of Millien [8] and Rinderknecht & Blanco [11]. The highest body mass estimates (700+ kg) calculated by those authors were calculated using anteroposterior dimensions: basicranial length, condylobasal length, diastema length, total skull length and rostral length (in order from highest to lowest mass estimate). Incisor width was also high in Rinderknecht and Blanco [11], but this may be due to primarily sampling large caviomorphs. Mass estimates using transverse dimensions (incisor width in Millien [8], frontal width) or dimensions of the masticatory apparatus (tooth row length, zygomatic length) were much lower, and in many cases were similar to the estimates produced here using log-allometric skull length or corrected OCW with paracondyles (approx. 350–500 kg). This might imply that *Josephoartigasia* has an overall elongate skull in the anteroposterior axis compared with smaller-bodied, extant rodents, which would agree with Cardini [41].

A similar issue with nonlinear allometry arises under OCW if log-allometry is not accounted for. Under OCW, body mass estimates under a log-linear all-taxon model, a log-allometric all-taxon model, and a log-allometric model including only rodents are all relatively similar (see electronic supplementary material). However, when predicting from a log-linear model created from only rodents, body mass estimates are much higher, particularly in the very largest taxon (*Josephoartigasia*), in which the log-linear, rodent-only model predicts a mass 80% higher than the log-allometric, all-taxon model and more than twice as large as the log-linear, all-taxon model (336.2 kg, (162.4–696.1 kg), versus 146–189 kg in other OCW models without correction factors; see electronic supplementary material, Information). Interestingly, comparing the present results with the body mass estimates of Vucetich *et al.* [13] shows a similar pattern: body mass estimates in Vucetich *et al.* [13] largely agree with one another, previous studies, and this study at smaller sizes. However, in the largest taxa considered (*Phoberomys* spp. and *Josephoartigasia monesi*) mass estimates begin to wildly diverge from one another, as is the issue among previous studies. This suggests that the assumption of geometric isometry used by Vucetich *et al.* [13] may not hold at large sizes, which would agree with the log-allometric relationship seen here. The present example gives an excellent case study of how the common assumption that log-transformation completely linearizes biological data may not always be accurate, and failing to account for nonlinear allometry can possibly lead to biologically spurious conclusions.

The primary issue with estimating body mass in extinct giant caviomorphs is one of extrapolation. Taxa like *Josephoartigasia* and *Phoberomys* are so much larger than any living rodent that it is challenging to reconstruct body size, especially with a rodent-only sample [8,17,18]. Millien [8] suggested that comparisons with a broader sampling of mammals, such as ungulates, would be necessary to resolve the issue of body size in these taxa. The present study accomplishes that: OCW is a metric that is found to closely correlate with body size across Theria [23] and comparisons using skull length are made here with a broader sample beyond just Rodentia. In both cases the dataset includes a broad enough sample of body sizes to avoid extrapolation. Dinomyids and neoepiblemids do appear to have narrower condyles than expected, but OCW can still be used to constrain body mass estimates, especially if differences in occiput shape are corrected for. Considering even the most extreme body mass estimates under OCW are dramatically lower than many of those previously reported in the literature, despite all sampled extant mammals (including *Dinomys branickii*) falling within this range of variation, previous estimates of body mass in excess of 600 kg for *Josephoartigasia monesi* and *Phoberomys pattersoni* are unlikely. Body masses of approximately 500 kg for *Josephoartigasia* and 150–200 kg for *Phoberomys* are better supported.

In the context of the present study, it is suggested that for dinomyids the most reliable body mass estimates are calculated from OCW including paracondyles assuming a similar degree of overestimation as *Dinomys*, which would result in a body mass of approximately 480–500 kg for *Josephoartigasia monesi*. For neoepiblemids, it is suggested the most appropriate of the regression models considered here is the OCW model treating this taxon as having ‘rabbit-like’ condyles, though the volumetric estimates of Kerber *et al.* [21] for *Neoepiblema* may be more accurate. This would produce estimates of approximately 125–150 kg for *Phoberomys pattersoni*. Notably, body mass estimates from skull length, HBL and shape-corrected OCW (with or without paracondyles) are largely similar to one another (around 500 kg for *J. monesi* and 150 kg for *P. pattersoni*), suggesting a general agreement in estimated body size. Giant chinchilloid caviomorphs like *Josephoartigasia* and *Phoberomys* were undoubtedly some of the largest and most impressive rodents to ever exist, but they were probably smaller in size (less than 600 kg) than initially estimated.

## Data Availability

All data and code used to produce this study is included in the electronic supplementary material [42].
